# Disease burden of mental disorders among children and adolescents considering both co-morbidities and suicide in Northeastern China

**DOI:** 10.1186/s12889-024-18721-5

**Published:** 2024-05-20

**Authors:** Yanxia Li, Qian Chen, Li Liu, Xing Yang, Huijuan Mu, Qihao Wang, Jian Lian, Huijie Chen, Yuan Gao, Lingjun Yan, Wei Sun, Guowei Pan

**Affiliations:** 1 Institute of Preventive Medicine, China Medical University, Shenyang, People’s Republic of China; 2 Institute of Chronic Diseases Liaoning Provincial Center for Disease Control and Prevention, Shenyang, People’s Republic of China; 3 Research Center for Universal Health, School of Public Health, China Medical University, No.77 Puhe Road, North New Area, Shenyang, 110122 People’s Republic of China

**Keywords:** Mental disorders, Children and adolescents, Co-morbidities, Suicide, DALYs

## Abstract

**Background:**

Few studies have assessed the burden of mental disorders among children and adolescents considering the impact of co-morbidities and suicide on disability adjusted life years (DALYs).

**Methods:**

This was a multicenter cross-sectional study. Our survey data in Liaoning Province (LN) were used to estimate the burden of six mental disorders, supplemented with data from other investigative studies conducted in China to assess four other disorders. DALYs were derived from the sum of years lived with a disability (YLDs) adjusted for co-morbidities, and the years of life lost (YLLs) adjusted for suicide. The changes in DALYs, YLDs, and YLLs were compared with and without adjustment for co-morbidities and suicide.

**Results:**

The DALYs rate of mental disorders among children and adolescents in LN decreased from 1579.6/10^5^ to 1391.4/10^5^, after adjusting for both co-morbidities and suicide (-11.9%). The DALYs rate for major depression, anxiety disorder, and conduct disorder (-80.8/10^5^, -75.0/10^5^ and  -30.2/10^5^, respectively) were the top three contributors to the DALYs reduction (-188.2/10^5^). The YLDs decreased from 72724.8 to 62478.5 after co-morbidity adjustment (-17.8%), mainly due to the reduction by major depression (-35.3%) and attention deficit/hyperactivity disorder [ADHD] (-34.2%). The YLLs increased from 130 to 1697.8 after adjusting for suicides (+ 56.9% of all suicide YLLs), mainly due to the contribution of major depression (+ 32.4%) and anxiety disorder (+ 10.4%). Compared to GBD 2010, the estimated DALY rate for mental disorders in LN was to be about 80%, with the proportion of DALYs and DALY rates explained by major depressive disorder accounted for only approximately one-third (14.6% vs. 41.9% and 202.6 vs. 759.9, respectively). But the proportion and absolute level of DALY rates explained by anxiety disorders were approximately 2-fold higher (39.7% vs. 19.6% and 552.2 vs. 323.3, respectively).

**Conclusions:**

The DALYs of mental disorders among Chinese children and adolescents were approximately 80% of the global level, with anxiety disorders imposing about 2 times the global level. Co-morbidity and suicide must be adjusted when calculating DALYs.

## Introduction

Global burden of disease (GBD) studies have shown that mental disorders are the leading cause for years lived with disability (YLDs) and the 7th most frequent cause for the disability adjusted life years (DALYs) [1, 2]. For individuals < 30 years of age, mental disorders account for 15-30% of DALYs [3]. The WHO estimated that if left untreated, the presence of mental health disorders during childhood may lead to up to 10-fold higher costs during adulthood [4].

According to our knowledge, only two studies have focused on DALYs globally due to mental disorders in children and adolescents based on GBD data. Erskine and colleagues (2015) estimated the global DALYs of children and youth 0–24 years of age were 61.8 million in 2010, and Piao et al. (2022) estimated the global DALYs of young people 0–19 years of age to be 21.5 million in 2019. The large difference between the two studies may be due to the following: (i) the inconsistency in age groups between the two studies, with the greatest burden of mental disorders among those 20–24 years of age; (ii) Piao et al. (2022) did not estimate DALYs due to substance use disorders; and (iii) unlike Erskine et al. (2015), Piao et al. (2022) did not adjust for co-morbidities and suicide.

As populous countries, China and India are estimated to account for approximately one-third of the global DALYs due to mental, neurologic, and substance use disorders, which is greater than all developed countries combines [5]. Anxiety is a common mental health problem in China. It has been reported that Chinese youths are more likely to have anxiety compared to counterparts in Western countries, where depression is predominant [6, 7]. The United Nations International Children’s Emergency Fund (UNICEF) has estimated that at least 30 million children and adolescents < 17 years of age in China struggle with emotional or behavioral problems [8]. Therefore, estimating the burden of mental disorders among children and adolescents in China is important from an epidemiologic and public health policy perspective.

No studies to date have focused on the burden of mental disorders among children and adolescents in China based on local survey data. The major challenges of conducting such a study are as follows: (i) There is limited population-representative prevalence data with a full range of mental disorders in China, especially among children and adolescents. Estimates from a previous GBD study were based on modeling projections with no data, and the final estimates reflected a wide range of uncertainty. (ii) Studies had a very narrow age range due for methodologic reasons [9]. In addition, the only report on the burden of mental disorder in China presented DALYs of the national level without adjusting co-morbidities and suicide [10]. Data at the provincial level can provide more valuable information and help inform local health resource policies in China.

It is worth noting that un-adjustment for co-morbidity and suicide deaths have a significant influence on the estimation of DALYs. Several studies have shown that co-morbidities associated with mental illnesses, if not considered, could lead to a 15-28% overestimation of YLDs [11–13], and un-considering suicide deaths could result in an underestimation of 59-62% of total suicide YLLs [5, 14]. Of the two studies of DALYs due to mental disorders involving children and adolescents, only Erskine and colleagues (2015) adjusted for co-morbidities for YLDs and attributed YLLs to suicide, and found an additional 6.3 million attributable suicidal YLLs globally. Erskine et al. (2015), however, did not quantify YLDs for unadjusted co-morbidities or assess the effects of co-morbidities.

The purpose of this study was as follows: (i) estimate DALYs in Liaoning Province (LN), China using local survey data on children and adolescents; (ii) adjust for co-morbidities and suicide, and assess the influence on disease burden; and (iii) compare the study findings with the GBD general level, and show the characteristics of Chinese children and adolescents.

## Methods

### Data sources

This was a multicenter cross-sectional study. To estimate DALYs of mental disorders in children and adolescents more accurately, we collected prevalence and mortality data. The prevalence data for this study were derived from three cross-sectional studies in China, the main one conducted on mental disorders among Chinese children and adolescents (6–17 years of age) living in 3 cities and 3 rural counties of LN by our team in 2008. A two-stage sampling procedure was used with the Strengths and Difficulties Questionnaire (SDQ) as the screening instrument and the Development and Well-being Assessment (DAWBA) as the diagnostic instrument [15, 16]. Of a total of 9806 students eligible for the screening phase, 434 who refused to participate, 74 who did not return the questionnaire and 810 who failed the questionnaire were excluded. Thus, self-reported valid SDQs were collected from 8488 students. Clinical assessment was conducted using the parent and adolescent versions of the DAWBA diagnostic interview, which used a mixture of closed and open questions about child psychiatric symptoms and their impact. According to Diagnostic and Statistical Manual of Mental Disorders fourth edition (DSM-IV), all the diagnoses were made by two experienced child psychiatrists who had completed the online training available for the DAWBA. The prevalence data of six common mental disorders, including anxiety disorders, major depression, dysthymia, attention deficit/hyperactivity disorder (ADHD), conduct disorder, and eating disorders, were obtained. Considering that the survey years and prevalence levels were similar and both surveys were conducted in China, the current study used data from Hunan Province to complement the low prevalence of schizophrenia and idiopathic intellectual disability based on DSM-IV [17]. With respect to alcohol and drug use disorders, we cited national survey data sorted by DSM-IV disorder categories, including LN [18]. Mortality and suicide data were retrieved from the Vital Registry System of Liaoning Provincial Center for Disease Control and Prevention (LNCDCP). The population size in LN was 4.6 million (6–17 years of age).

### DALYs

DALYs referred to the total years of healthy life lost from onset-to-death, and was calculated by summing the YLDs and YLLs (DALY = YLL + YLD). One DALY was defined as the loss of a healthy life-year.

The YLLs were calculated from the number of deaths multiplied by a standard life expectancy at the age at which death occurred:

YLL = N*L,

Where N was the number of deaths due to a cause of death in a given age group or gender and.

L was the life expectancy value corresponding to the age group in a standard life table.

A YLD was a measure of the amount of life lost due to disability. YLDs per person from a sequela were equal to the prevalence of the sequela multiplied by the disability weight for the health state associated with that sequela. YLDs of a disease were the sum of the YLDs of each sequela associated with the disease. In this study the severity of distribution and disability weight were referred to the GBD study.

YLD was calculated as the prevalence of each non-fatal condition multiplied by its disability weight.

YLD sequela = P × DW.

YLD=∑YLD sequela.

P was the number of prevalent cases for one cause and DW was the disability weight.

### Co-morbidities

A combined disability weight was required to account for individuals with co-morbidities. To calculate a combined disability weight, the health loss associated with two disability weights were multiplied together, then a weighted average of each constituent disability weight was calculated. In this study only the co-morbidities between six mental disorders based on our survey data were considered.

$${DW}_{adj}=1-{\prod }_{s}(1-{DW}_{s})$$

To allocate disability among health states, DW_adj_ was proportionally redistributed to these health states:

$${DW}_{adj,s}={DW}_{adj}*\frac{{DW}_{s}}{\sum _{s=1}^{Z}{DW}_{s}}$$

The adjusted YLD was then calculated as:

$${YLD}_{adj}=\sum _{s=1}^{z}{n}_{s}*{DW}_{adj,s}$$

### Attributable burden

To quantify the burden of suicide attributable to mental disorders, we conducted supplementary comparative risk assessment (CPA) analyses [19, 20]. The association between mental disorders and suicide was well-recognized with the relative risk (RR) ranging from 2.7 (95% uncertainty interval [UI] = 1.7–4.3) for an anxiety disorder and 19.9 (95% UI = 9.5–41.7) for a major depressive disorder [20]. Population attributable fractions (PAFs) were determined from the prevalence of exposure to each disorder and the RR for suicide. Finally, these PAFs were multiplied by the corresponding suicide YLLs to estimate suicide burden attributable to mental disorders by gender and age. The methodology and process for calculating the proportion of suicide burden attributable to the disorders has been described in detail elsewhere [19, 20].

## Results

Table 1 showed the prevalence of mental disorders by gender and age group in LN, including survey data on anxiety disorders, major depression, dysthymia, ADHD, conduct disorder, and eating disorders from our study [15, 16], with additional supplemental data on the prevalence of schizophrenia and idiopathic intellectual disability from the investigative literature in Hunan Province [17], as well as alcohol and substance use disorders from a national study in five provinces [18].

Table 2 showed a comparison of YLDs, YLLs, DALYs, and DALY rates before and after adjusting for co-morbidities and suicide of each mental disorders in LN. Overall, the DALYs and DALY rates were 64176.3 and 1391.4/10^5^, respectively, after adjustment, with a reduction of 11.9%. Anxiety disorders (25467.2), major depression (9342.8), conduct disorder (8575.9) and eating disorder (4937.8) were the top four disorders for DALYs, accounting for 39.7%, 14.6%, 13.4% and 7.7% of the total DALYs, respectively. The DALYs rate for major depression, anxiety disorder, and conduct disorder (-80.8/10^5^, -75.0/10^5^ and  -30.2/10^5^, respectively) were the top three contributors to the DALYs reduction (-188.2/10^5^). The YLDs decreased from 72724.8 to 62478.5 after co-morbidity adjustment (-17.8%), mainly due to the reduction by major depression (-35.3%) and ADHD (-34.2%). Anxiety disorders, conduct disorder, major depression and eating disorders were the top four disorders of YLDs. After including suicides, the YLLs increased by 1697.8 YLLs, equivalent to 56.9% of all suicide YLLs. Of the suicide DALYs attributable to mental disorders, major depression was responsible for the largest proportion (+ 32.4%), followed by anxiety disorder (+ 10.4%), alcohol use disorder (+ 9.3%), eating disorders (+ 4.6%), and schizophrenia (+ 0.2%).

**Table 1:** Prevalence (%) of mental disorders in children and adolescents by gender and age group

Children and Adolescents mental disorders	Males			Females		
	6 ∼ 9	10 ∼ 14	15 ∼ 17	6 ∼ 9	10 ∼ 14	15 ∼ 17
Anxiety disorders	2.779	10.175	6.204	0.547	6.514	7.158
	(2.429, 3.129)	(9.532, 10.818)	(5.691, 6.717)	(0.390, 0.704)	(5.989, 7.039)	(6.610, 7.706)
Major depression	0.001	1.655	2.278	0.001	0.515	3.038
	(0, 0.008)	(1.384, 1.926)	(1.961, 2.595)	(0, 0.008)	(0.363, 0.667)	(2.673, 3.403)
Dysthymia	0.001	0.001	0.027	0.001	0.001	0.001
	(0, 0.008)	(0, 0.008)	(0, 0.062)	(0, 0.008)	(0, 0.008)	(0, 0.008)
ADHD	0.189	1.970	1.093	0.273	0.001	0.194
	(0.097, 0.281)	(1.674, 2.266)	(0.872, 1.314)	(0.162, 0.384)	(0, 0.008)	(0.100, 0.288)
Conduct disorder	1.524	3.208	2.402	0.273	1.825	0.291
	(1.263, 1.785)	(2.833, 3.583)	(2.076, 2.728)	(0.162, 0.384)	(1.540, 2.110)	(0.176, 0.406)
Eating disorders	0.001	0.154	0.108	0.091	1.516	0.985
	(0, 0.008)	(0.071, 0.237)	(0.038, 0.178)	(0.027, 0.155)	(1.256, 1.776)	(0.775, 1.195)
Schizophrenia	0.009	0.009	0.016	0.011	0.010	0.019
	(0, 0.028)	(0. 0.028)	(0, 0.041)	(0, 0.032)	(0, 0.030)	(0, 0.047)
Idiopathic intellectual disability	1.123	1.029	1.965	1.330	1.218	2.327
	(0.911, 1.335)	(0.826, 1.232)	(1.686, 2.244)	(1.100, 1.560)	(0.997, 1.439)	(2.024, 2.630)
Alcohol use disorder	0.068	0.667	2.294	0.031	0.232	0.795
	(0.049, 0.087)	(0.608, 0.726)	(2.186, 2.402)	(0.018, 0.044)	(0.197, 0.267)	(0.731, 0.859)
Drug use disorders	0.084	0.679	2.340	0.023	0.220	0.749
	(0.063, 0.105)	(0.620, 0.738)	(2.231, 2.449)	(0.012, 0.034)	(0.186, 0.254)	(0.687, 0.811)
Other mental disorders	0.094	2.806	0.001	0.925	0.677	0.888
	(0.029, 0.159)	(2.455, 3.157)	(0, 0.008)	(0.721, 1.129)	(0.503, 0.851)	(0.688, 1.088)

Note: The prevalence of schizophrenia, idiopathic intellectual disability, alcohol use disorders and substance use disorders by gender and age group was adjusted for the overall prevalence characteristics of the survey population in the original study

**Table 2:** Comparison of YLDs, YLLs, DALYs and DALY rates (per 100,000) before and after adjustment

Children and Adolescents mental disorders	ICD10	YLDs before comorbidity adjustment	YLDs after comorbidity adjustment	Difference in YLDs	Changes in YLDs(%)	YLLs before suicide attribution	YLLs after suicide attribution	Difference in YLLs	As a proportion of all suicide YLLs (%)	DALYs (before)	DALYs (after)	Difference in DALYs	Changes in DALYs(%)	DALY rates (before)	DALY rates (after)	Difference in DALY rates
**Anxiety disorders**	**F40-F44.9, F93-F93.2**	**28927.6** **(26623.3, 31233.8)**	**25181.7** **(24001.7, 27065.3)**	**-3745.9** **(-4168.6, -2621.6)**	**-12.9%**	**0**	**285.5** **(265.2, 305.5)**	**+ 285.5** **(265.2, 305.5)**	**10.4%** **(9.6%, 11.1%)**	**28927.6** **(26623.3, 31233.8)**	**25467.2** **(24266.9, 27370.8)**	**-3460.4** **(-3863.1, -2356.4)**	**-12.0%**	**627.2** **(577.2, 677.2)**	**552.2** **(526.1, 593.4)**	**-75.0** **(-83.8, -51.1)**
**Major depression**	**F32-F33.9**	**13068.0** **(11098.3, 15052.5)**	**8451.7** **(7367.0, 9450.0)**	**-4616.3** **(-5602.5, -3731.3)**	**-35.3%**	**0**	**891.1** **(810.7, 965.0)**	**+ 891.1** **(810.7, 965.0)**	**32.4%** **(29.5%, 35.1%)**	**13068.0** **(11098.3, 15052.5)**	**9342.8** **(8177.7, 10415.0)**	**-3725.2** **(-4637.5, -2920.6)**	**-28.5%**	**283.3** **(240.6, 326.4)**	**202.6** **(177.3, 225.8)**	**-80.8** **(-100.5, -63.3)**
**Dysthymia**	**F34.1**	**34.6** **(21.1, 79.5)**	**34.6** **(21.1, 79.5)**	**0**	**0**	**0**	**--**	**-**	**-**	**34.6** **(21.1, 79.5)**	**34.6** **(21.1, 79.5)**	**0**	**0**	**0.8** **(0.5, 1.7)**	**0.8** **(0.5, 1.7)**	**0**
**ADHD**	**F90-F90.9**	**401.4** **(317.2, 482.7)**	**264.0** **(215.1, 305.7)**	**-137.4** **(-177.0, -102.1)**	**-34.2%**	**0**	**-**	**-**	**-**	**401.4** **(317.2, 482.7)**	**264.0** **(215.1, 305.7)**	**-137.4** **(-177.0, -102.1)**	**-34.2%**	**8.7** **(6.9, 10.5)**	**5.7** **(4.7, 6.6)**	**-3.0** **(-3.8, -2.2)**
**Conduct disorder**	**F91-F92.9**	**9969.8** **(8443.6, 11414.6)**	**8575.9** **(7502.5, 9907.7)**	**-1393.9** **(-1506.9, -941.1)**	**-14.0%**	**0**	**-**	**-**	**-**	**9969.8** **(8443.6, 11414.6)**	**8575.9** **(7502.5, 9907.7)**	**-1393.9** **(-1506.9, -941.1)**	**-14.0%**	**216.2** **(183.1, 247.5)**	**185.9** **(162.7, 214.8)**	**-30.2** **(-32.7, -20.4)**
**Eating disorders**	**F50-F50.9**	**5161.8** **(3912.1, 6371.3)**	**4809.0** **(3648.8, 5916.0)**	**-352.8** **(-455.3, -263.3)**	**-6.8%**	**0**	**128.8** **(100.9, 156.0)**	**+ 128.8** **(100.9, 156.0)**	**4.6%** **(3.7%, 5.7%)**	**5161.8** **(3912.1, 6371.3)**	**4937.8** **(3749.7, 6072.0)**	**-224.0** **(-299.3, -162.5)**	**-4.3%** **(-4.7%, -4.2%)**	**111.9** **(84.8, 138.1)**	**107.1** **(81.3, 131.6)**	**-4.9** **(-6.5, -3.5)**
**Schizophrenia**	**F20-F20.9, F25-F25.9**	**401.8** **(0, 1114.2)**	**401.8** **(0, 1114.2)**	**-**	**-**	**0**	**5.7** **(0, 13.9)**	**+ 5.7** **(0, 13.9)**	**0.2%** **(0, 0.5%)**	**401.8** **(0, 1114.2)**	**407.5** **(0,1128.1)**	**+ 5.7** **(0, 13.9)**	**+ 1.4%**	**8.7** **(0, 24.2)**	**8.8** **(0, 24.5)**	**+ 0.1** **(0,0.3)**
**Idiopathic intellectual disability**	**F70-F79**	**3781.5** **(3167.7, 4394.5)**	**3781.5** **(3167.7, 4394.5)**	**-**	**-**	**0**	**-**	**-**	**-**	**3781.5** **(3167.7, 4394.5)**	**3781.5** **(3167.7, 4394.5)**	**0**	**0**	**82.0** **(68.7, 95.3)**	**82.0** **(68.7, 95.3)**	**0**
**Alcohol use disorder**	**F10.2**	**3422.8** **(3173.1, 3671.3)**	**3422.8** **(3173.1, 3671.3)**	**-**		**0**	**256.7** **(242.1, 271.1)**	**+ 256.7** **(242.1, 271.1)**	**9.3%** **(8.8%, 9.9%)**	**3422.8** **(3173.1, 3671.3)**	**3679.5** **(3415.2, 3942.4)**	**+ 256.7** **(242.1, 271.1)**	**+ 7.5%**	**74.2** **(68.8, 79.6)**	**79.8** **(74.0, 85.5)**	**+ 5.6** **(5.2, 5.9)**
**Other drug use disorder**		**3900.5** **(3617.6, 4182.6)**	**3900.5** **(3617.6, 4182.6)**	**-**		**0**	**-**			**3900.5** **(3617.6, 4182.6)**	**3900.5** **(3617.6, 4182.6)**	**0**	**0**	**84.6** **(78.4, 90.7)**	**84.6** **(78.4, 90.7)**	**0**
**Other mental disorders**		**3655.0** **(3011.8, 4302.7)**	**3655.0** **(3011.8, 4302.7)**	**-**	**-**	**130.0** **(0, 384.8)**	**130.0** **(0, 384.8)**	**-**	**-**	**3785.0** **(3011.8, 4687.5)**	**3785.0** **(3011.8, 4687.5)**	**0**	**0**	**82.1** **(65.3, 101.6)**	**82.1** **(65.3, 101.6)**	**0**
**All mental disorders**	**F01-F99**	**72724.8** **(63385.9, 82299.6)**	**62478.5** **(55726.4, 70389.4)**	**-10246.3** **(-11910.3, -7659.5)**	**-17.8%**	**130.0** **(0, 384.8)**	**1697.8** **(1418.9, 2096.3)**	**+ 1567.8** **(1418.9, 1711.5)**	**56.9%** **(51.6%, 62.2%)**	**72854.8** **(63385.9, 82684.4)**	**64176.3** **(57145.3, 72485.6)**	**-8678.5** **(-10198.8, -5859.9)**	**-11.9%**	**1579.6** **(1374.3, 1792.7)**	**1391.4** **(1239.0, 1571.6)**	**-188.2** **(-221.1, -135.3)**

Note: Changes in DALY rates (%) were the same as that of DALYs. Eating disorders were inclusive of anorexia nervosa and bulimia nervosa. Only anorexia nervosa was estimated when calculating the attributable suicide YLLs.

**Fig. 1:**
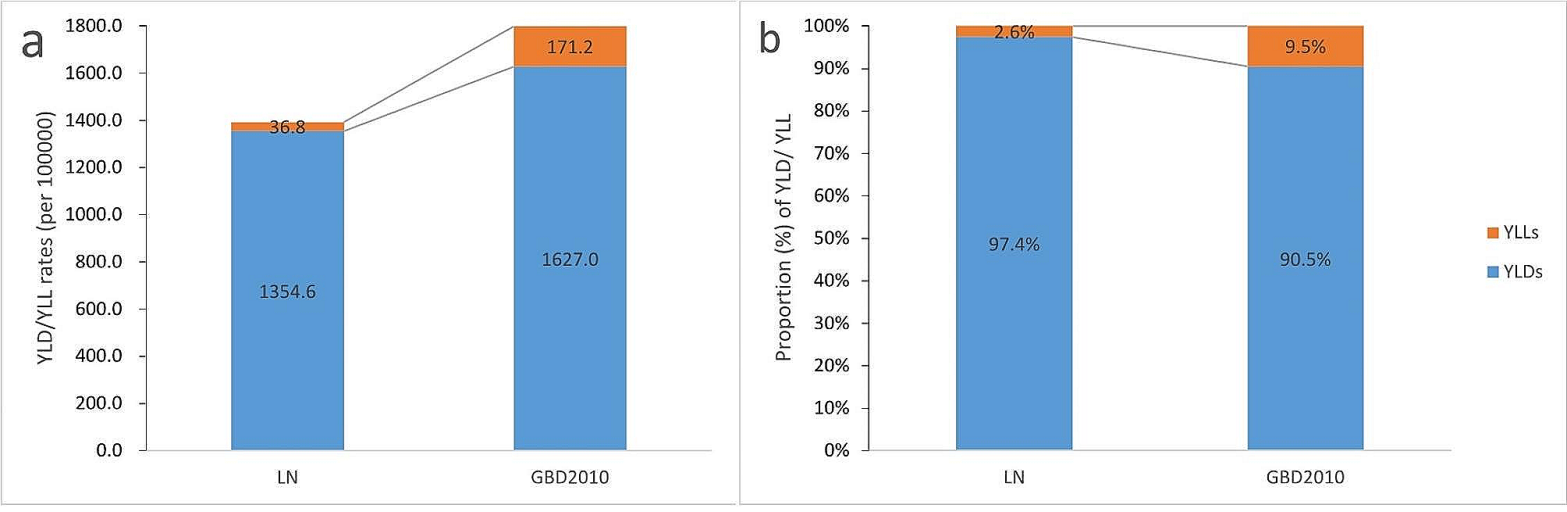
Absolute levels (**a**) and proportions (**b**) of YLDs/YLLs after adjustment between LN and GBD 2010

Figure 1 compared the corresponding absolute levels (a) and proportions (b) of YLDs/YLLs of all mental disorders after adjustment for co-morbidity and suicide between LN and GBD 2010 (5–19 year age group). Figure 1-a showed significantly lower rates of YLDs and YLLs in LN(1354.6 and 36.8, respectively)than GBD (1627.0 and 171.2, respectively), as well as the ratio of YLDs and DALYs (2.6% and 9.5%, respectively) between LN and GBD in Fig. 1-b.

**Fig. 2:**
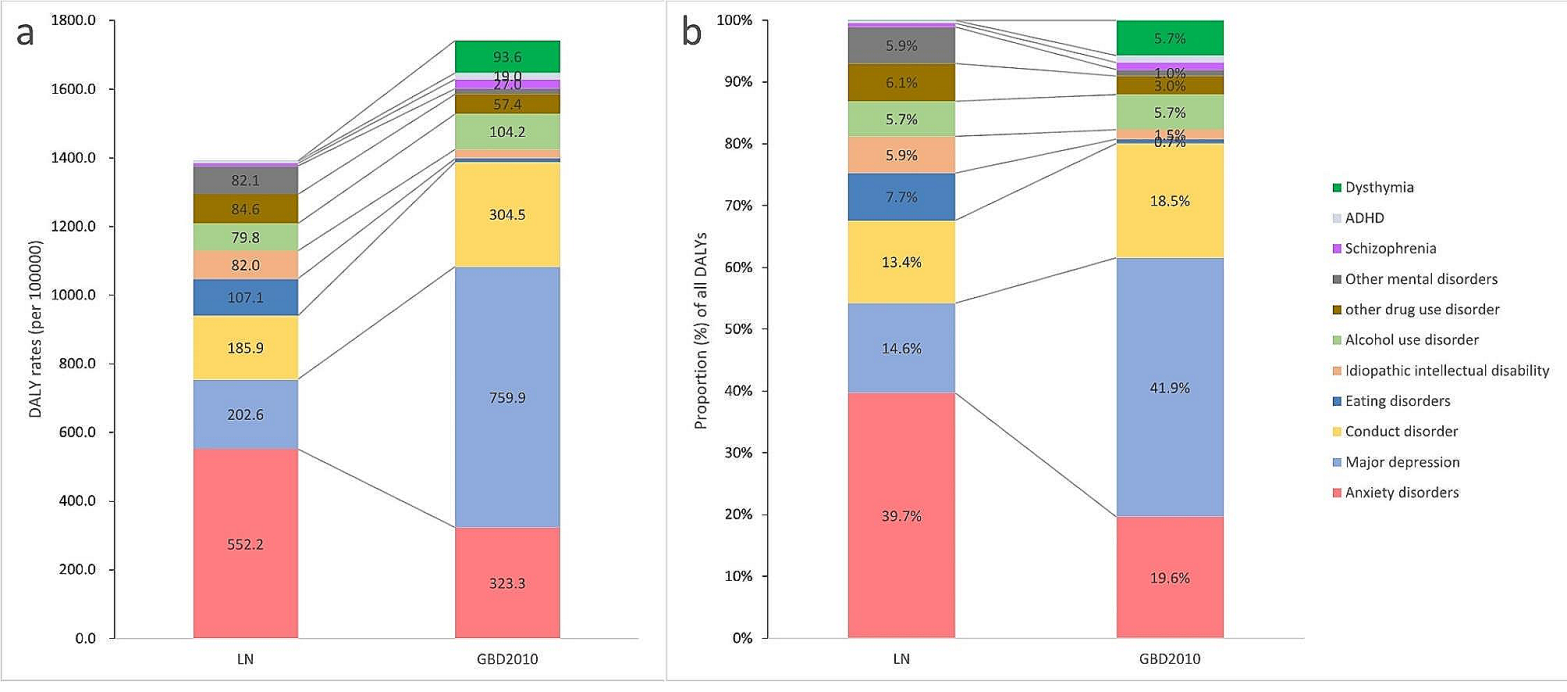
Absolute levels (**a**) and proportions (**b**) of DALYs per disorder between LN and GBD 2010

The absolute levels (a) and proportions (b) of DALY rates for each mental disorder between LN and GBD 2010 in the 5–19 year age group after adjustment for co-morbidity and suicide were summarized in Fig. 2. The DALY rate for mental disorders among children and adolescents in LN was estimated to be about 80% of the global estimate. Although anxiety and major depressive disorders together accounted for > 50% of the total, when compared to GBD 2010 the proportion and absolute level of DALY rates explained by anxiety disorders in LN were approximately 2-fold higher (39.7% vs. 19.6% and 552.2 vs. 323.3, respectively), but the proportion of DALYs and DALY rates explained by major depressive disorder accounted for only approximately one-third (14.6% vs. 41.9% and 202.6 vs. 759.9, respectively).

The GBD 2010 data were obtained from the GBD 2010 Results by Cause 1990–2010 (https://ghdx.healthdata.org/record/ihme-data/gbd-2010-results-cause-1990-2010), and suicide YLLs data were from the GBD 2010 study [20].

## Discussion

It has been estimated that the DALY rate of mental disorders in LN was 1391.4 per 100,000 in children and adolescents after adjustment for co-morbidities and suicide. According to our knowledge, this is the first report on the mental burden of children in China. The burden of Chinese children was approximately 80% of GBD, which may reflect the relatively lower level of mental disorder prevalence and suicide mortality in Chinese children and adolescents [21, 22]. Like the previous findings [13, 19, 20], we found similar changing patterns of burden levels and structure, decreased DALYs and YLDs, and increased YLLs. No adjustment will result in a 17.8% overestimation of YLDs, a 56.9% underestimation of suicide YLLs, and a 11.9% overestimation of DALYs in LN, suggesting the significance of two adjustments in the DALYs estimation.

As expected, we found a significantly different structure of DALYs between LN and GBD 2010. The 2-fold high burden of anxiety disorders in LN reflected the distinct pattern of mental disorders in Chinese children, with similar finding observed in the Chinese study, although not adjusted for co-morbidity and suicide [10]. The significantly higher anxiety disorder level could be related to the industrialization and urbanization in China over the past 40 years, as well as the increasing pressure and competition faced at school, the high expectations of parents, and the rapidly changing socioeconomic conditions, all of which can make Chinese children and adolescents more anxious than children in other countries [7, 23, 24]. Interestingly, although anxiety disorders were responsible for the majority of DALYs, the change in the YLDs rate caused by co-morbidities and the proportion of suicide-associated YLLs were both lower than YLDs and YLLs attributed to major depression. This finding suggests that a healthier environment, especially for parents and schools, should be created to prevent more children and adolescents from developing anxiety, as well as major depression, which is more harmful and even more likely to incite suicide, and requires continuous mental care and effective intervention in key populations in China.

In this study adjusting for co-morbidities resulted in an overall decrease in YLDs (17.8%), which is comparable to the 15-28% decrease reported in the previous studies [11–13]. The current study not only considered co-morbidity between six mental disorders based on our survey data, which may also have led to an underestimation of co-morbid YLDs. Our study also showed that the most commonly co-occurring psychiatric disorders were associated with ADHD and major depression, although ADHD was responsible for the lowest DALYs. ADHD has also been reported to have a high level of association with co-morbidities and other mental disorders [25, 26]. The co-morbidities associated with other psychiatric disorders, especially depression, anxiety, and conduct disorder in childhood, are substantial [27, 28]. Shared genes have been also found in ADHD, conduct disorder, and major depression, and the symptoms often change concurrently [29, 30]. The low DALY rate of ADHD may be related to the fact that the Chinese may be constitutionally less vulnerable to ADHD on the basis of a more placid inborn temperament. Co-morbidities can lead to a more complicated and severely symptomatic presentation of each disorder, and more attention should be paid to these disorders, especially ADHD and depression, to facilitate better mental health outcomes [28, 31].

Although the link between mental disorders and suicide is well-documented [32, 33], fewer mental disorders are recognized as a primary cause of death in mortality registrations, as this typically involves the cumbersome task of disentangling the effect of multiple mental and physical disorders, which leads to an underestimation of YLLs [20, 34]. The results of reattribution analyses provide a more comprehensive insight into the magnitude of the burden due to these disorders. Previous GBD studies have shown that mental and substance use disorders are responsible for 59 − 62% of suicide YLLs across the globe [19, 20], which is comparable to our results. One possible explanation is that the prevalence of major depression in LN is lower than the global level, and the relative risk of suicide for major depression is the highest. In addition, we found that the proportion of total suicide-associated YLLs attributed to major depression was the highest, followed by anxiety and alcohol use disorders. The ranking is basically in agreement with the global ranking [20]. These findings also underscore the importance of prioritizing prevention, early detection, and effective management of mental disorders, especially major depressive disorder, as key strategies for reducing YLLs in China.

## Limitation

Several study limitations need to be acknowledged. First, the DALYs estimation was inevitably limited by a lack of data, especially for children and adolescents. In the current study, our data accounted for 60% of the DALYs estimation and the other 40% of the prevalence data citing other provincial or national studies to substitute, which will inevitably introduce some biased estimation. With respect to the prevalence of diseases, approximately 80% diseases from our survey data, we are of the opinion that the estimates are reasonable and acceptable. Second, the survey data utilized in this study contained approximately 10% missing data attributable to refusals or exclusions, which may increase uncertainty about the prevalence and thus limit the estimation of YLDs to some extent. Third, because of the limitation in detailed co-morbidity data, only co-morbidities between several mental disorders in our data were considered, which can also lead to an over- or under-estimation of the final YLDs. Fourth, we estimated the suicide-associated YLLs to the burden based on mental disorders, for which evidence of excess mortality and sufficient relative risk data for suicide were available, but which may not reflect the burden of suicide causing other psychiatric disorders, such as conduct disorder and ADHD, that tend to underestimate mental disorder YLLs. Despite these limitations, we conclude that the estimation of DALYs was reliable because we have covered most disorders and age groups with all the data available and made two adjustments.

## Conclusion

The burden of mental disorders among Chinese children and adolescents was approximately 80% of the global level. But structurally, the burden of anxiety disorders was approximately 2-fold the global estimate, and only one-third was accounted for by major depression. Prioritizing prevention, early identification, and effective management of mental disorders, particularly anxiety disorder, are warranted for reducing the mental disorder burden in Chinese children and adolescents. Co-morbidity and suicide must be adjusted when calculating DALYs, otherwise the DALYs will be overestimated.

## Data Availability

The datasets generated and analyzed during the current study are not publicly available because of our agreement with the participants, but are available from the corresponding author on reasonable request.
